# Notification that new names of prokaryotes, new combinations and new taxonomic opinions have appeared in volume 75, part 2 of the IJSEM

**DOI:** 10.1099/ijsem.0.006728

**Published:** 2025-05-30

**Authors:** Aharon Oren, Markus Göker

**Affiliations:** 1 The Institute of Life Sciences, The Hebrew University of Jerusalem, The Edmond J. Safra Campus, 9190401 Jerusalem, Israel; 2 Leibniz Institute DSMZ – German Collection of Microorganisms and Cell Cultures, Inhoffenstrasse 7B, 38124 Braunschweig, Germany

This listing of names of prokaryotes published in a previous issue of the *IJSEM* is provided as a service to microbiology to assist in the recognition of new names and new combinations. This procedure was proposed by the Judicial Commission [1]. The names given herein are listed according to the Rules of priority (i.e. the time and date of publication of the articles on the journal’s platform and the order of valid publication of names in the original articles) [2].

**Table:** 

Order of article publication	Name/author	Proposed as	Article no.	Page no.
1	*Tersicoccus mangrovi* Sibero *et al*. 2025	sp. nov.	006669	6
2	*Acidovorax sacchari* Sawada *et al*. 2025	sp. nov.	006575	9
3	*Campylobacter molothri* Miller *et al*. 2025	sp. nov.	006635	8
4	*Lacrimispora sinapis* Ren *et al*. 2025	sp. nov.	006675	5
5	*Streptococcus wuxiensis* Zhou *et al*. 2025	sp. nov.	006674	7
5	*Streptococcus jiangnanensis* Zhou *et al*. 2025	sp. nov.	006674	7
5	*Streptococcus fermentans* Zhou *et al*. 2025	sp. nov.	006674	8
6	*Tamlana flava* Huang *et al*. 2025	sp. nov.	006673	8
6	*Allotamlana* Huang *et al*. 2025	gen. nov.	006673	9
6	*Cognatitamlana* Huang *et al*. 2025	gen. nov.	006673	9
6	*Neotamlana* Huang *et al*. 2025	gen. nov.	006673	10
6	*Pseudotamlana* Huang *et al*. 2025	gen. nov.	006673	10
6	*Allotamlana fucoidanivorans* (Li *et al*. 2020)Huang *et al*. 2025	comb. nov. [basonym: *Tamlana fucoidanivorans* Li *et al*. 2020]	006673	10
6	*Cognatitamlana onchidii* (Yin *et al*. 2021) Huang *et al*. 2025	comb. nov. [basonym: *Algibacter onchidii* Yin *et al*. 2021]	006673	10
6	*Neotamlana sedimentorum* (Romanenko *et al*. 2014) Huang *et al*. 2025	comb. nov. [basonym: *Tamlana sedimentorum* Romanenko *et al*. 2014]	006673	10
6	*Neotamlana nanhaiensis* (Liu *et al*. 2025) Huang *et al*. 2025	comb. nov. [basonym: *Tamlana nanhaiensis* Liu *et al*. 2025]	006673	10
6	*Neotamlana laminarinivorans* (Li *et al*. 2023) Huang *et al*. 2025	comb. nov. [basonym: *Tamlana laminarinivorans* Li *et al*. 2023]	006673	10
6	*Neotamlana sargassicola* (Li *et al*. 2023) Huang *et al*. 2025	comb. nov. [basonym: *Tamlana sargassicola* Li *et al*. 2023]	006673	11
6	*Pseudotamlana agarivorans* (Yoon *et al*. 2008) Huang *et al*. 2025	comb. nov. [basonym: *Tamlana agarivorans* Yoon *et al*. 2008]	006673	11
6	*Pseudotamlana carrageenivorans* (Jung *et al*. 2019) Huang *et al*. 2025	comb. nov. [basonym: *Tamlana carrageenivorans* Jung *et al*. 2019]	006673	11
6	*Pseudotamlana haliotis* (Cao *et al*. 2021) Huang *et al*. 2025	comb. nov. [basonym: *Tamlana haliotis* Cao *et al*. 2021]	006673	11
7	*Oceanobacter antarcticus* Zhang *et al*. 2025	sp. nov.	006676	7
8	*Afipia dichlorophenoxyacetatis* Sawada *et al*. 2025	sp. nov.	006672	10
9	*Minisyncoccus* Nakajima *et al*. 2025*	gen. nov.	006668	12
9	*Minisyncoccus archaeiphilus* Nakajima *et al*. 2025	sp. nov.	006668	12
9	*Minisyncoccaceae* Nakajima et al. 2025	fam. nov.	006668	12
9	*Minisyncoccales* Nakajima *et al*. 2025	ord. nov.	006668	12
9	*Minisyncoccia* Nakajima *et al*. 2025	class. nov.	006668	13
9	*Minisyncoccota* Nakajima *et al*. 2025	phyl. nov.	006668	13
10	*Methanobacterium aridiramus* Lee *et al*. 2025	sp. nov.	006658	6
11	*Thalassobellus* Lee *et al*. 2025	gen. nov.	006663	9
11	*Thalassobellus suaedae* Lee *et al*. 2025	sp. nov.	006663	9
12	*Pseudoalteromonas holothuriae* Yvin *et al*. 2025	sp. nov.	006601	6
13	*Roseicyclus amphidinii* Cui *et al*. 2025	sp. nov.	006682	7
14	*Pseudoalteromonas ardens* Thomas *et al*. 2025	sp. nov.	006681	9
14	*Pseudoalteromonas obscura* Thomas *et al*. 2025	sp. nov.	006681	10
14	*Pseudoalteromonas umbrosa* Thomas *et al*. 2025	sp. nov.	006681	10
15	*Flavobacterium fructosi* Han *et al*. 2025	sp. nov.	006694	6
15	*Flavobacterium xylosi* Han *et al*. 2025	sp. nov.	006694	6
15	*Flavobacterium zhoui* corrig. Han *et al*. 2025†	sp. nov.	006694	6
16	*Vogesella oryzagri* Huq *et al*. 2025	sp. nov.	006687	8
17	*Novosphingobium aquae* Hong *et al*. 2025	sp. nov.	006688	6
17	*Novosphingobium anseongense* Hong *et al*. 2025	sp. nov.	006688	9
18	*Antrihabitans spumae* Suescún-Sepúlveda *et al*. 2025	sp. nov.	006695	9

*The protologue gives species type instead of type species.

†The list editors have corrected the epithet *zhouii* to *zhoui* in accordance with the new guidelines of Appendix 9 of the ICNP that took effect on 3 January 2025 [3].

The following taxonomic opinions (i.e. the emendation of circumscriptions or the creation of synonyms) do not affect the status of a name under the International Code of Nomenclature of Prokaryotes. These taxonomic opinions can neither be considered as approved nor as disapproved by the International Committee on Systematics of Prokaryotes.

**Table:** 

Name/author	Article no.	Page no.
*Actinomadura catellatispora* Lu *et al*. 2003 pro synon. *Actinomadura livida* Lavrova and Preobrazhenskaya 1975 (Approved Lists 1980)*	006684	7
*Actinomadura livida* Lavrova and Preobrazhenskaya 1975 (Approved Lists 1980) emend. Dif *et al*. 2025	006684	9
*Antrihabitans* Lee *et al*. 2020 emend. Suescún-Sepúlveda *et al*. 2025	006695	9
*Treponema pallidum* (Schaudinn and Hoffman 1905) Schaudinn 1905 (Approved Lists 1980) emend. Norris 2025	006697	4

*The authors gave Avrova instead of Lavrova.
